# Changes in children respiratory infections pre and post COVID-19 pandemic

**DOI:** 10.3389/fcimb.2025.1549497

**Published:** 2025-04-07

**Authors:** Yuanyuan Yue, Dan Wu, Qian Zeng, Yurong Li, Chun Yang, Xin Lv, Ling Wang

**Affiliations:** ^1^ Clinical Laboratory, Children’s Hospital Affiliated to Shandong University, Jinan, China; ^2^ Clinical Laboratory, Jinan Children’s Hospital, Jinan, China

**Keywords:** COVID-19, respiratory infection, non-pharmaceutical interventions, children, qPCR

## Abstract

**Background:**

Non-pharmaceutical interventions (NPIs) implemented during the COVID-19 pandemic had a significant impact on the prevalence of various acute respiratory infections (ARIs) pathogens.

**Methods:**

We collected 337,310 real-time PCR results for 13 pathogens from clinical samples between January 2018 and January 2024 to assess the changes of ARIs among children before and after the COVID-19 pandemic.

**Results:**

A variety of ARIs pathogens, including Influenza A (Flu A), Influenza B (Flu B), Adenovirus (ADV), Rhinovirus (RhV), and Respiratory Syncytial Virus (RSV), as well as co-infecting bacterial such as Klebsiella pneumoniae (KPN), Pseudomonas aeruginosa (PAE), Streptococcus pneumoniae (SP), Haemophilus influenzae (HI), and Legionella pneumophila (LP), reached a peak positive rate at the age of 3. The susceptible age of Mycoplasma pneumoniae (MP) was from 3 to 7 years old. Compared to the pre-COVID pandemic period, the positive rates of Flu A, MP, ADV, SP, HI, *Staphylococcus aureus* (SA) and KPN decreased during the COVID-19 pandemic. And the positive rates of Flu B and PAE increased. Compared to the period during the COVID-19 pandemic, the positive rates of Flu A, ADV, RSV, RhV, SP, HI, KPN, PAE and SA were increased after the pandemic. Conversely, the positive rates of MP, Flu B, and Parainfluenza virus (PIV) decreased.

**Conclusions:**

The implementation of NPIs interrupted the circulation of ARIs pathogens. However, release of NPIs and the reduced baseline of population immunity, may contribute to a resurgence of ARIs pathogens among children.

## 1 Introduction

Influenza virus, RSV and RhV are the three leading viral pathogens and SP, MP and KPN are the three leading bacterial pathogens in China between 2009eenns (Li et al., 2021). Common childhood respiratory pathogens, including MP, Flu A, Flu B, ADV, RSV, PIV and RhV through aerosol particles and respiratory droplets during close contact (Li et al., 2022; Meyer Sauteur et al., 2022). The general population is susceptible to these infections, which manifest epidemically every few years, often reaching their peak in winter around January or February (Olsen et al., 2021). Furthermore, many bacteria also cause of childhood respiratory invasive infections, include KPN, KPN, SA, SP, HI, LP. The rates of these respiratory bacterial diseases are influenced by the frequency of bacterial exposure and host susceptibility (Major et al., 2023). Epidemiological, clinical, and experimental evidence suggests that certain viruses can enhance susceptibility to bacterial respiratory infections (Tsang et al., 2020; Palani et al., 2023). Given that SARS-CoV-2 is a respiratory virus, the COVID-19 pandemic may increase susceptibility to respiratory bacterial infections (Danino et al., 2022; Lubkin et al., 2024).

Since the outbreak of COVID-19, Jinan city in Shandong province entered a stage of normalization epidemic prevention and control. These robust non-pharmaceutical interventions (NPIs) not only reduce the spread of COVID-19 but also influence the epidemiology of common respiratory pathogens in children to some extent (Spinelli et al., 2021; Cai et al., 2024). The spread and seasonality of these respiratory pathogens have been temporarily interrupted, and the incidence of childhood bacterial infectious respiratory disease also changed (Perra, 2021; Meyer Sauteur et al., 2022). On December 7, 2022, China ended its “Zero‐COVID” policy (Zhou et al., 2023). This transition allowed for a restoration of normalcy in people’s lives. Following this, a significant rise in Omicron cases emerged in China, accompanied by a sharp increase in influenza incidence in February 2023 (Chakraborty et al., 2023). Additionally, other pathogens causing respiratory tract infections appeared successively during this period. Although people still keep maintain certain NPI habits, such as maintaining social distance, washing hand frequently, and wearing face mask.

We gathered a total of 337,310 Real-time Quantitative polymerase chain reaction (qPCR) results for KPN, PAE, SA, SP, HI, LP, MP, Flu A, Flu B, ADV, RSV, PIV, and RhV from clinical samples collected between January 2018 and January 2024 in Children’s Hospital Affiliated to Shandong University to investigate the epidemiological features of prevalent respiratory pathogens in children both prior to and following the COVID-19 pandemic.

## 2 Methods

### 2.1 Data collection

This study was approved by the Ethics Committee of Children’s Hospital Affiliated to Shandong University (Ethical approval number: SDFE-IRB/P-202315). The requirement for informed consent was waived because this study was based on a retrospective analysis of electronic medical records (EMRs). Children with respiratory tract infections, either inpatient or outpatient, were enrolled between January 1, 2018, and January 31, 2024. Inclusion criteria were as follows: 1) diagnosed with respiratory infection; 2) PCR results are available. Exclusion criteria were as follows: 1) children with severe malformations, including large atrial or ventricular septal defects, bronchopulmonary dysplasia, dextrocardia, and neuromuscular diseases; 2) children diagnosed with malignant tumors or primary immunodeficiency diseases, or those who received immunosuppressive drugs during hospitalization.

### 2.2 Temporal segmentation

On January 20, 2020, the National Health Commission declared that COVID-19 pneumonia would be classified as a Category B infectious disease according to the law of the People’s Republic of China regarding the Prevention and Treatment of Infectious Diseases, which had received approval from the State Council, and they implemented prevention and control strategies applicable to Category A infectious diseases. In response to the continuing COVID-19 pandemic, Shandong province implemented a first-level response for Major Public Health Emergency on January 24, 2020. The majority of these preventive measures were non-pharmaceutical in nature and comprised the blockade of areas affected by the outbreak, border closures, limitations on travel, restrictions on social and public activities, shutdowns of schools and businesses, stay-at-home directives, promotion of physical distancing, an emphasis on hand hygiene practices, and the use of face masks. On December 7, 2022, the State Council of China issued the “Notice on Further Optimizing the Implementation of COVID-19 prevention and control measures”. Cancel mass nucleic acid testing and health code inspection (except in special places); allow mild and asymptomatic infections to be isolated at home; no longer delineate high-risk areas, narrow the scope of sealing and control. Those marking a major adjustment of the COVID-19 pandemic prevention and control policy from “dynamic Zero‐COVID” to “scientific and precise prevention and control”. Subsequently, on January 8, 2023, the Chinese government announced that COVID-19 would downgrading Class B diseases from enhanced to routine management. The focus has shifted to health resource preparation, vaccination and protection of vulnerable populations. Therefore, we period from January 1, 2018, to December 31, 2019, has been designated as the pre-COVID-19 pandemic phase, from January 1, 2020, to December 31, 2022, has been designated as during the COVID-19 pandemic phase; and from January 1, 2023, to January 31, 2024, has been designated as post-COVID-19 pandemic phase.

### 2.3 Specimens collection and detection

ARIs samples comprised nasopharyngeal swabs, pharyngeal swab, sputum, bronchoalveolar lavage fluid and pleural effusion, were collected by trained nurses according to Standard Operating Procedures (SOP). These samples were promptly transported to the clinical laboratory, tested by the Respiratory Pathogen Nucleic Acid Diagnostic Detection Kit (Beijing Zhuocheng Huisheng Biotechnology Co. Ltd and SANSURE BIOTECH INC.), conducted by professional staff in adherence to established SOP. All test items were strictly subjected to quality control in accordance with the sample instructions. Thirteen ARIs pathogens were identified, including KPN, PAE, SA, SP, HI, LP, MP, Flu A, Flu B, ADV, RSV, PIV and RhV.

### 2.4 Statistical analysis

The overall detection rates of thirteen common pathogens, including KPN, PAE, SA, SP, HI, LP, MP, Flu A, Flu B, ADV, RSV, PIV, and RhV, were analyzed across three periods: prior to, during, and following the COVID-19 pandemic. To explore seasonal variations in viral activity and age-related differences in pathogen infections, the trends in detection rates from 2018.01 to 2024.01 and age-specific differences in pathogen infections were illustrated using GraphPad Prism 8.0. We performed chi-square analyses to assess changes in pathogen distribution across different phases of the COVID-19 pandemic in the **Supplementary Tables** (P<0.05).

## 3 Results

### 3.1 Study population

**Figure 1:**
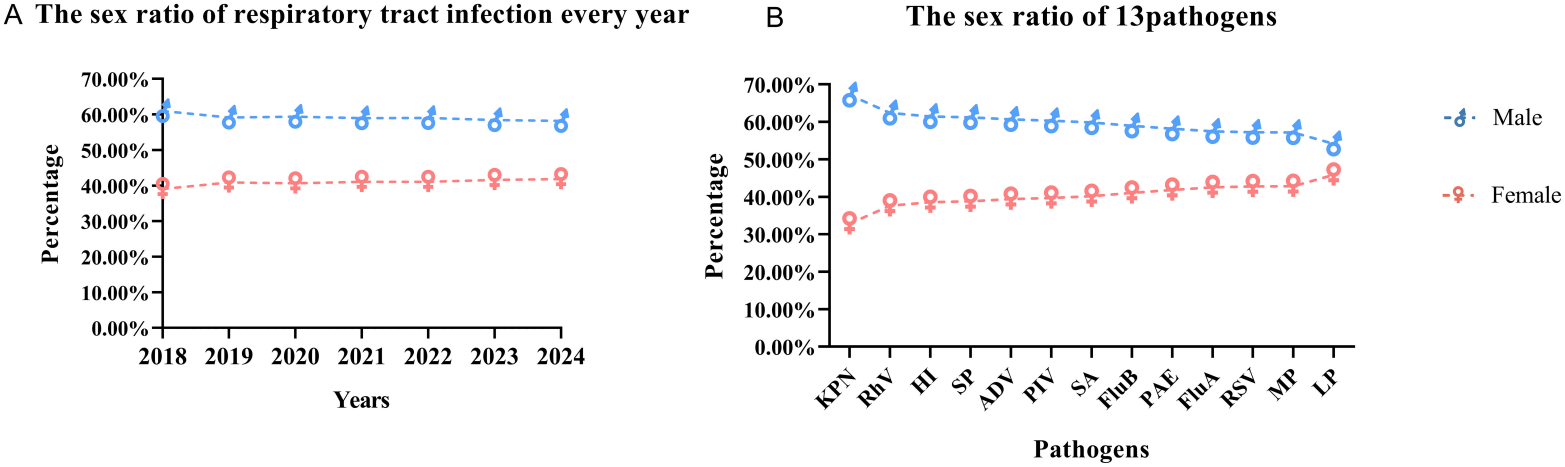
Sex distribution of pathogen positive results. **(A)**: The sex ratio of respiratory tract infection every year; **(B)**: The sex ratio of 13 pathogens.

**Figure 2:**
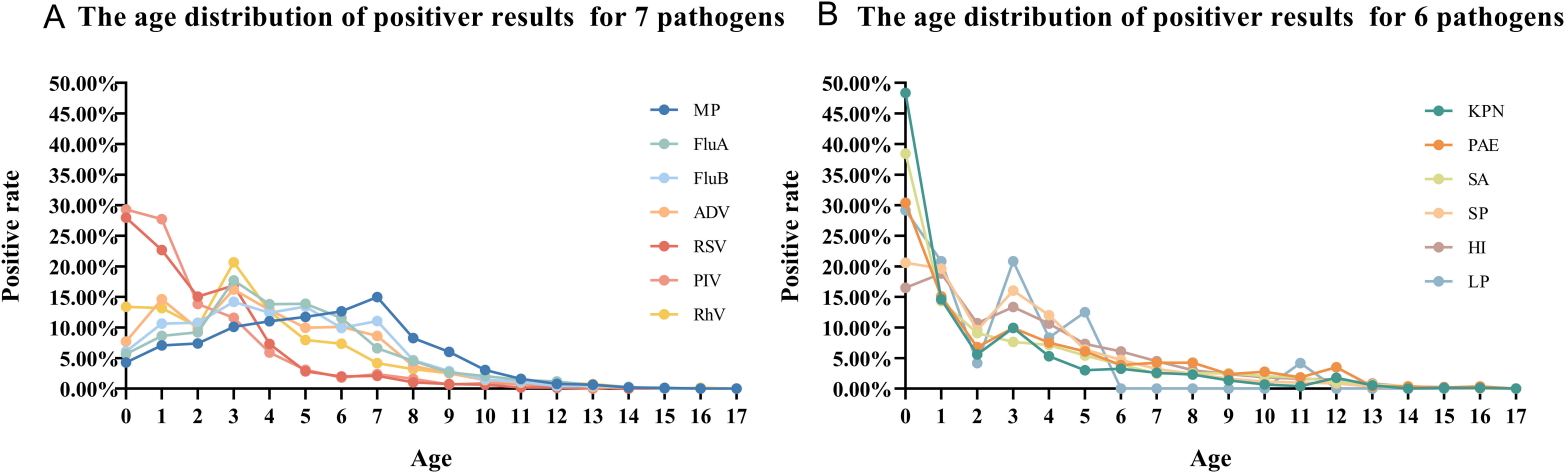
Age distribution of positive rates for 13 pathogens. **(A)**: The age distribution of positiver results for 7pathogens; **(B)**: The age distribution of positiver results for 6 pathogens.

A total of 337,310 children were included, the male-to-female ratio was 1.43:1, males (58.85%) were 17% higher than females (58.85%) (**Table 1**; **Figure 1A**). The gender distribution among the 13 pathogens was different, PAE was the highest (2.05), LP was the lowest at 1.18, (**Figure 1B**). All children were divided into five age groups: 0~1 y (17.20%), 1~3 y (25.24%), 3~6 y (33.52%), and ≥6 y (24.04%) (**Table 1**). The positive rates of eight pathogens (KPN, PAE, SA, SP, HI, LP, RSV and PIV) were highest at age 0~1, gradually decreased with age. The positive rates of four pathogens(Flu A, Flu B, ADV and RhV) were highest at age 3 and MP’s positive rate was highest at age 7 at 14.97% (**Figure 2**).

**Table 1:** The PCR results of 13 pathogens were summarized from January 31, 2018 to January 31, 2024.

year		2018 N=2761 n %		2019 N=22785 n %		2020 N=27337 n %		2021 N=71093 n %		2022 N=113049 n %		2023 N=82332 n %		2024 N=17953 n %		Sum N=337310 n %	
sex	male	1684	60.99	13472	59.13	16224	59.35	41893	58.93	66679	58.98	48093	58.41	10446	58.19	198491	58.85
	female	1077	39.01	9313	40.87	11113	40.65	29200	41.07	46370	41.02	34239	41.59	7507	41.81	138819	41.15
age	0-1	1375	49.80	4511	19.80	5622	20.57	12526	17.62	20786	18.39	10809	13.13	2398	13.36	58027	17.20
	1-3	836	30.28	6577	28.87	8671	31.72	18814	26.46	27703	24.51	18517	22.49	4007	22.32	85125	25.24
	3-6	342	12.39	7304	32.06	8026	29.36	22293	31.36	41574	36.78	29202	35.47	4328	24.11	113069	33.52
	>6	208	7.53	4393	19.28	5018	18.36	17460	24.56	22986	20.33	23804	28.91	7220	40.22	81089	24.04
pathogen	KPN	83	6.01	83	4.53	37	2.76	99	1.24	91	1.49	320	8.84	21	5.48	734	3.24
	PAE	25	1.81	26	1.42	27	2.01	148	1.85	104	1.70	168	4.64	45	11.75	543	2.40
	SA	182	13.19	227	12.38	119	8.87	465	5.83	412	6.73	282	7.79	17	4.44	1704	7.52
	SP	414	30.00	525	28.64	283	21.09	1344	16.84	963	15.72	2205	60.91	290	75.72	6024	26.58
	HIB	354	25.65	546	29.79	256	19.08	1749	21.92	1164	19.00	1008	27.85	138	36.03	5215	23.01
	LP	1	0.07	2	0.11	7	0.52	4	0.05	5	0.08	5	0.14	0	0.00	24	0.11
	MP	62	4.49	1757	16.30	518	5.55	5269	19.93	1375	5.83	258	2.40	125	9.45	9364	11.20
	Flu A	-	-	777	22.14	966	20.69	7	0.13	1727	10.57	2587	18.72	116	3.70	6180	13.13
	Flu B	-	-	143	4.10	674	14.46	1399	25.05	1619	9.91	66	0.48	595	18.96	4496	9.56
	ADV	-	-	303	9.53	170	4.62	450	4.22	1191	7.13	987	7.31	580	18.94	3681	7.25
	RSV	-	-	-	-	298	10.70	868	18.38	1221	7.84	1842	13.41	805	25.65	5034	12.60
	PIV	-	-	-	-	45	5.72	405	4.78	309	5.78	107	2.67	14	0.57	880	4.18
	RhV	-	-	-	-	16	20.25	240	14.65	1474	11.28	1184	13.06	146	11.04	3060	12.16

“n”: Number of positives; “%”: Positive rate.

KPN, Klebsiella pneumoniae; PAE, Pseudomonas aeruginosa; SA, Staphylococcus aureus; SP, Streptococcus pneumoniae; HI, Haemophilus influenzae; LP, Legionella pneumophila; MP, Mycoplasma Pneumoniae; Flu A, Influenza A virus; Flu B, Influenza B virus; ADV, Adenoviridae; RSV, respiratory syncytial virus; PIV, parainfluenza virus; RhV, Rhinovirus; “-”: The test was not performed in our laboratory.“-”

### 3.2 Monthly changes in PCR results for 7 infectious pathogens from 2018.01 to 2024.01

Before the COVID-19 pandemic, PCR was only used for MP testing, and we established PCR testing for Flu A, Flu B and ADV in June 2019, with the increased application of PCR testing during the COVID-19 pandemic, PCR testing for RSV in January 2020, PIV in July 2020 and RhV in December 2020 were established.

From January 31, 2018, to January 31, 2024, we collected a total of 314,648 respiratory tract infectious samples. These samples were not tested for all seven pathogens(MP, Flu A, Flu B, ADV, RSV, PIV and RhV) simultaneously. Some specimens were individually tested for single pathogen such as Mp, while others underwent combination tests (Flu A/Flu B/RSV, ADV/PIV/RhV, MP/Flu A/Flu B, or Flu A/Flu B/RSV/ADV/PIV).

**Figure 3:**
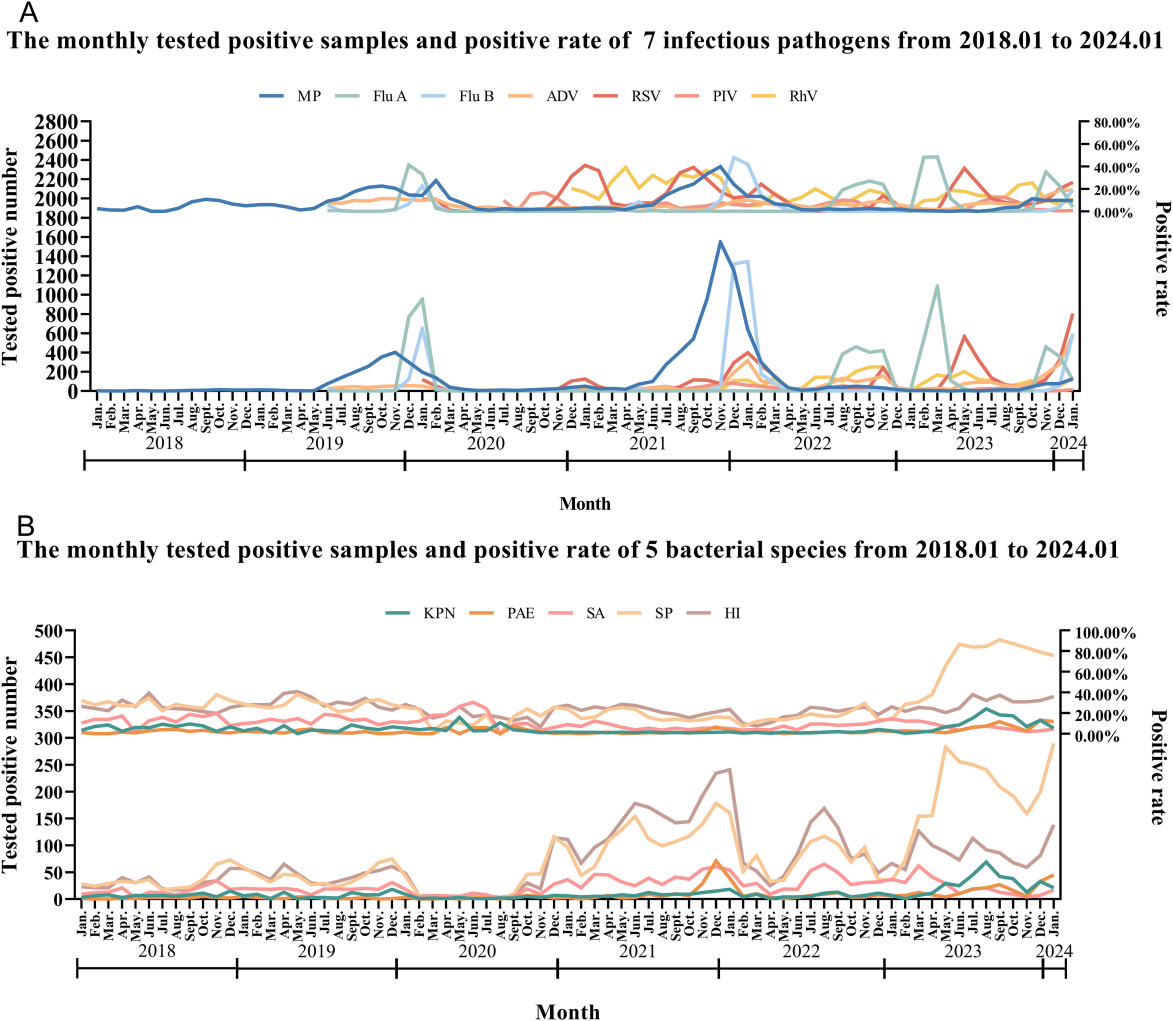
The monthly positive samples of testing systems and test positive rate of 12 infectious pathogens from 2018.01 to 2024.01. **(A)**: The monthly tested positive samples and positive rate of 7 infectious pathogens from 2018.01 to 2024.01; **(B)**: The monthly tested positive samples and positive rate of 5 bacterial species from 2018.01 to 2024.01.

From January 31, 2018, to January 31, 2024, We detected two peaks of MP epidemics (2019.07~2020.03, 2021.07~2022.02), with the monthly positive rate was greater than 11.52%, and the highest was 39.80%. Four peaks of Flu A epidemics (2019.12~2020.01, 2022.08~2022.11, 2023.02~2023.04, 2023.11~2023.12), with the monthly positive rate was greater than 11.07%, the highest was 48.44%. Two epidemic peaks of Flu B occurred in January 2020 and from December 2021 to February 2022, with the monthly positive rate was greater than 14.29% and the highest was 47.71%. Two epidemic peaks of AVD (2019.10~2020.02, 2023.10~2024.01), with the monthly positive rate was greater than 9.70%, and the highest was 18.94%. RSV had six epidemic peaks scattered from January 2020 to January 2024, with the monthly positive rate was greater than 10.27%, the highest positive rate was 40.98%. PIV had two epidemic peaks (2020.09~12, 2023.07~08), with the monthly positive rate was greater than 11.11% and the highest positive rate was 16.67%. The RhV epidemic peak was recorded from March to November 2021, with the monthly positive rate exceeding 26.67% and a maximum positive rate of 39.13%. (**Figure 3A**; **Supplementary Table 1**). However, the positive number for both PIV and RhV remained relatively low throughout the year.

### 3.3 Monthly changes in PCR results for 5 bacterial species from 2018.01 to 2024.01

From January 31, 2018, to January 31, 2024, we collected a total of 22,662 respiratory tract infection samples, each of these samples underwent PCR testing for six types of bacteria: KPN, PAE, SA, SP, HI, and LP. Since the positive rate of LP was too low (24/22662, 0.11%), we no longer analyze the data for this bacterium.

Before the COVID-19 pandemic, between January 2018 and December 2019, the positive number and positive rates for the five bacteria (KPN, PAE, SA, SP, and HI) remained relatively stable. The positive rates were 5.17% at KPN, 1.59% at PAE, 12.73% at SA, 29.23% at SP, and 28.01% at HI. With the outbreak of COVID-19 in 2020, the number of positive cases and the positive rates of five bacterial strains decreased significantly, however, the order of positive rates remained relatively consistent compared to the pre-COVID-19 period. From January to December 2020, the total number of respiratory tract specimens was 1,342, the positive rates were 2.76% at KPN, 2.01% at PAE, 8.87% at SA, 21.09% at SP, and 19.08% at HI. During the mid-to-late stages of the COVID-19 pandemic, particularly after December 2020, the number of respiratory tract specimens for five bacterial experienced a significant rise, surpassing pre-COVID-19 pandemic levels, while the positive rates of five bacterial still lower than in the pre-COVID-19 pandemic period. From January 2021 to December 2022, the total number of respiratory tract specimens was 14,104, the positive rates were 1.35% at KPN, 1.79% at PAE, 6.22% at SA, 16.36% at SP, and 20.65% at HI. Notably, an infectious peak was observed in December 2021, with the largest increases in positive samples occurring in SP (187/1,100) and HI (234/1,100). From February to May 2022, the number of respiratory tract specimens decreased, followed by an increase from June to August, where the most significant rise in positive samples was again noted in SP and HI. After the peak in August, the number of specimens declined until January 2023, which may be attributed to summer vacation activities. From January 8, 2023, the control measures for COVID-19 were lifted. Between January 2023 and January 2024, we collected 4,003 respiratory tract specimens, with the positive rates were recorded as 8.52% for KPN, 5.32% for PAE, 7.47% for SA, 62.33% for SP, and 28.63% for HI. The most significant increase was observed at SP, with a positive rate of 62.33% (**Figure 3B**; **Supplementary Table 2**).

### 3.4 Changes in the positive rate of pathogens before and after the COVID-19 epidemic

**Figure 4:**
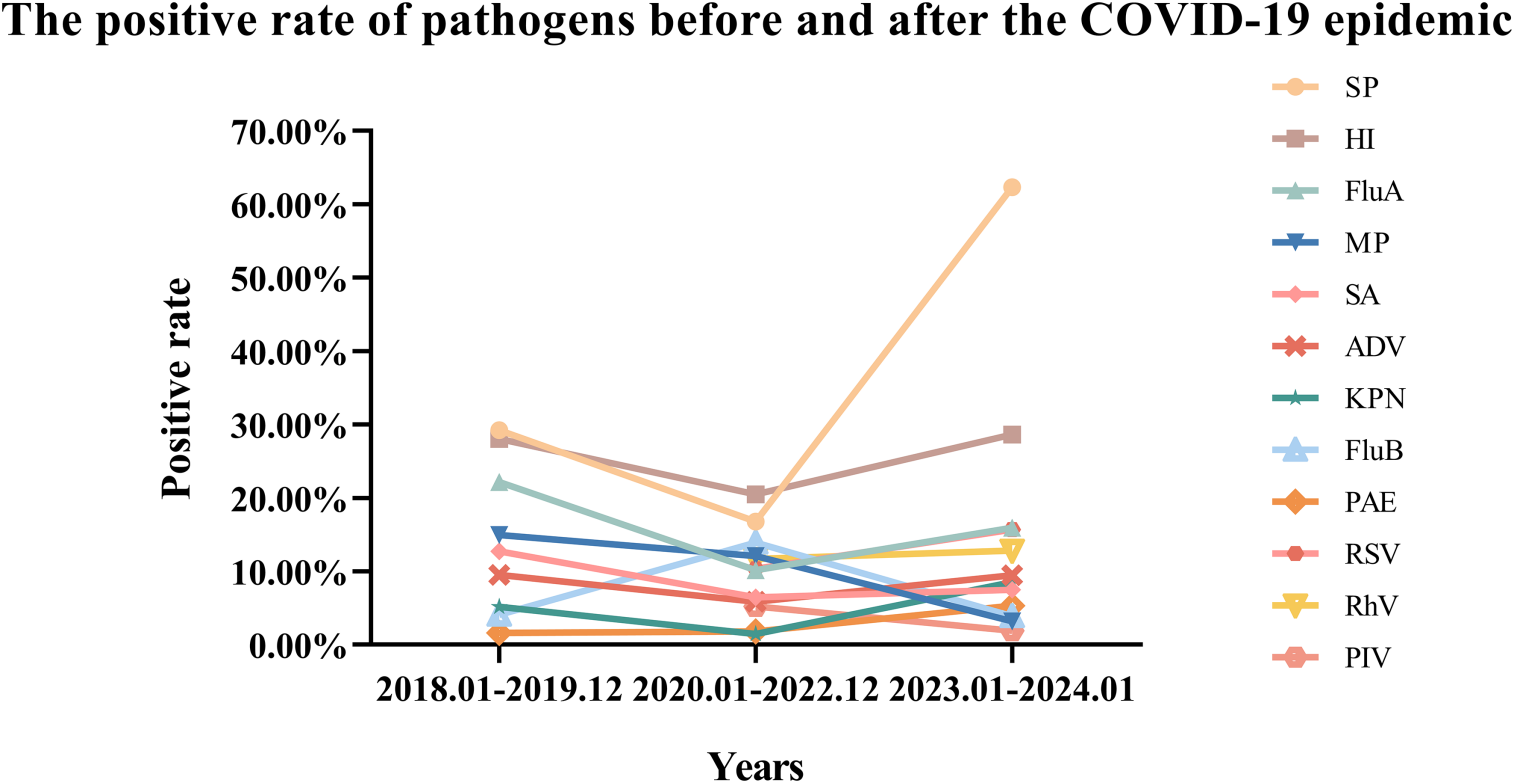
Changes in the positive rate of 12 pathogens before and after the COVID-19 epidemic.

Before the COVID-19 pandemic, the positive rates of five bacteria in respiratory tract specimens from high to low were SP, HI, SA, KPN, PAE. During the COVID-19 pandemic, the positive rates of these five bacteria from high to low were HI, SP, SA, PAE, and KPN. Following the COVID-19 pandemic, the positive rates of five bacteria from high to low were SP, HI, KPN, SA, and PAE. In comparison to the pre-COVID pandemic period, the positive rates of the four bacteria, excluding PAE, showed a decline during the COVID-19 pandemic. Specifically, SP decreased by 12.48% (P=0.000), HI by 7.52% (P<0.001), SA by 6.29% (P<0.001), and KPN by 3.70% (P<0.001) (**Supplementary Table 4**). However, after the COVID-19 pandemic, the positive rates of all five bacteria increased compared to the COVID-19 pandemic period, with SP increased by 45.58% (P=0.000), HI by 8.14% (P<0.001), SA by 1.03% (P=0.021), KPN by 7.05% (P<0.001), and PAE by 3.52% (P<0.001) (**Supplementary Table 4**). Notably, the most significant change in positive rate was observed in SP (**Figure 4**).

Before the COVID-19 pandemic, the positive rates of infectious pathogens from high to low were Flu A, MP, ADV, Flu B. During the COVID-19 pandemic, the positive rates of infectious pathogens from high to low were Flu B, MP, RhV, RSV, Flu A, ADV and PIV. After COVID-19 pandemic, the positive rates for infectious pathogens from high to low were Flu A, RSV, RhV, ADV, Flu B, MP, and PIV. Compared to the pre-COVID-19 period, the positive rates of three infectious pathogens decreased: Flu A decreased by 11.98%, MP decreased by 2.89%, ADV decreased by 3.69%, while Flu B increased by 9.78% during the COVID-19 pandemic. In comparison to the period during the COVID-19 pandemic, the positive rates of all four infectious pathogens increased after the COVID-19 pandemic: Flu A increased by 5.79% (P<0.001), ADV increased by 3.62% (P<0.001), RSV increased by 5.34% (P<0.001), RhV increased by 1.10% (P=0.009), however, the positive rates of the other three infectious pathogens decreased, as MP by 8.90% (P<0.001), Flu B by 9.99% (P<0.001), and PIV by 3.32% (P<0.001) (**Figure 4**) (**Supplementary Table 3**).

## 4 Discussion

Since the outbreak of SARS-CoV-2 in December 2019, China has implemented a series of NPIs to address this public health emergency (Yang et al., 2021; Leung et al., 2023). In February 2023, a sharp increase in Flu A activity, combined with the spread of the Omicron variant, posed a significant threat to the public healthcare system for children (Goldberg et al., 2023). To understand how the transmission dynamics of other respiratory viral infections co-evolve with those of SARS-CoV-2, we conducted a retrospective observation of the co-circulation of multiple respiratory pathogens at Children’s Hospital Affiliated to Shandong University from January 31, 2018, to January 31, 2024.

The sex ratio (male to female) of children with respiratory tract infections was approximately 1.43, while the sex ratio for individuals aged 0-19 years was 1.15, according to data from the National Bureau of Statistics (NBS) of China over the past five years. This disparity may be attributed to regional differences; additionally, it could be related to boys being more active and thus more likely to come into contact with sources of infection. Furthermore, studies indicate that girls’ immune systems develop earlier than boys’, leading females to produce stronger immune responses and antibodies to combat infectious pathogens (Oertelt-Prigione, 2012; Moulton, 2018; Sparks et al., 2023). Among five bacterial species studied, the highest sex ratio (2.05) was observed in KPN, while the lowest sex ratio (1.18) was found in LP. Among the seven infectious pathogens examined, RhV exhibited the highest sex ratio (1.66), whereas Flu A, RSV, and MP showed similar sex ratios of 1.34. Flu A, Flu B, ADV, RhV and RSV, as well as co-infecting bacterial pathogens such as KPN, PAE, SP, HI and LP, exhibit a peak positive rate at the age of 3. Children typically enter kindergarten at the age of 3, where close gatherings for learning and living can facilitate the spread of epidemic diseases. In contrast to viruses and bacteria, MP exhibits a broader age susceptibility, with positive rates exceeding 10% between the ages of 3 and 7, peaking at 14.97% at age 7.

In northern China, the period from November to February marks the peak season for epidemic respiratory diseases1. Prior to the COVID-19 pandemic, outbreaks of such diseases were identified from November 2019 to February 2020, including MP, Flu A, Flu B, ADV and RSV. With the NPIs implemented, from March 2020 to March 2021, many epidemic respiratory pathogens, including MP, Flu A, Flu B and ADV, circulated at low levels, and the incidence of various co-infecting bacteria, such as KPN, PAE, SA, SP and HI, was significantly reduced.3 However, during the winter of 2020, unusual epidemics of RSV and PIV emerged, which were not influenced by the NPIs11. On May 5, 2020, Jinan reduced its epidemic response level, leading to an increase in people’s activities and the reopening of schools. From July 2021 to February 2022, a resurgence of multiple infectious respiratory pathogens, including RhV, RSV, Flu B, and MP. Concurrently, the positive rates of co-infecting bacteria, specifically SP and HI, also showed a slight increase3. From February 2023 to January 2024, outbreaks of Flu A, ADV, RSV, PIV, and RhV, with a notable increase in the prevalence of SP among coinfectious bacteria.

In comparison to the period prior to the COVID-19 pandemic (January 2018 to December 2019), the positive rates of various infectious respiratory pathogens decreased during the pandemic (January 2020 to December 2022). This includes pathogens such as MP, Flu A, and ADV (Data for RSV, PIV and RhV were not available before the COVID-19). Additionally, the positive rates of co-infecting bacteria, including KPN, SA, SP and HI, also declined. This reduction may be attributed to the sustained implementation of NPIs, such as personal protective measures and physical distancing. Conversely, the positive rates of Flu B and PAE increased. In comparison to the COVID-19 pandemic period (January 2020 to December 2022), the positive rates of various infectious respiratory pathogens increased following the conclusion of the COVID-19 pandemic (January 2023 to January 2024). This includes pathogens such as Flu A, ADV, RSV, RhV. The positive rates of coinfected bacteria also increased, including KPN, PAE, SA, SP and HI. Conversely, the positive rates of MP and Flu B decreased. These changes in the transmission dynamics of respiratory pathogens may be attributed to modifications in COVID-19 pandemic mitigation measures, as well as shifts in prevalence and immunity. Meanwhile, this increase is attributed to the “immunity debt” resulting from the relaxation of COVID-19 pandemic restrictions.

Notably, lower levels of population immunity, particularly among younger children, could signal the potential for more widespread disease and a possibly more severe epidemic when influenza virus circulation resumes (Cai et al., 2024; Li et al., 2024).Investigations revealed that this increase was primarily due to the relaxation of COVID-19 control measures, which coincided with the onset of the cold season (Burrell et al. 2023; Meyer Sauteur et al.; Parums, 2023). After the epidemic ended, many children who had been unexposed to these pathogens for an extended period became more vulnerable (Cheng et al. 2022; Fujita, 2021).

In our study, we acknowledge several limitations, including the use of a single-center design, lack of detailed symptom severity data, underreporting, and potential bias introduced by the development of testing methods. To address these limitations, we plan to conduct more in-depth research in the future. Specifically, we will:1)Multi-center Data Collection: Collaborate with other medical institutions or research centers to jointly collect and analyze a larger dataset.2)Symptom Severity Data: Perform a retrospective case review to gather detailed information about symptom severity, design additional questionnaires or interviews, and directly inquire with patients or their parents about symptoms.3)Reduce Underreporting: Conduct community surveys to better understand children’s medical behaviors and health status during the epidemic. We will also explore other data sources (e.g., school absentee records, pharmacy sales data) to estimate the actual infection rate more accurately. Additionally, we will strengthen public health education and encourage children with mild symptoms to seek medical attention promptly.4)Consider the Impact of Testing Policy Changes: During data analysis, we will clearly distinguish between different time periods, particularly at points when testing policies changed. Statistical methods (e.g., breakpoint regression analysis) will be applied to assess the effect of these changes on the results. These efforts will require additional manpower, resources, and funding, which are part of our future research goals, though they are beyond our current capacity.

## 5 Conclusion

The results of this study suggest that the implementation of NPIs against COVID-19 likely restricted the transmission of Flu A, Flu B ADV and MP in children, and the incidence of co-infection bacterial pathogens was substantially reduced too. As China fully exited its “Zero‐COVID” policy, the co-circulation of multiple respiratory pathogens and increasing co-infection rates during the autumn and winter of 2023 indicate that respiratory viruses are not exhibiting typical seasonal circulation patterns, and a resumption of circulation of certain respiratory viruses is occurring. The retention of some NPIs post-COVID-19, such as improved hand hygiene, respiratory etiquette, physical distancing, and the use of masks in healthcare settings, may help mitigate the burden of infections caused by multiple respiratory pathogens. Increasing testing for multiple respiratory pathogens, promoting vaccine uptake, and enhancing non-pharmaceutical interventions can actively prevent the transmission of various respiratory pathogens in children.

## Data Availability

The original contributions presented in the study are included in the article/**Supplementary Material**. Further inquiries can be directed to the corresponding author.

## Supplementary material

Supplementary Table 1 Monthly results for 7 infectious pathogens from 2018.01 to 2024.01.

Supplementary Table 2 Monthly results for 5 bacterial species from 2018.01 to 2024.01.

Supplementary Table 3 Chi-square test for positive rate of 7 infectious pathogens before and after COVID-19 pandemic.

Supplementary Table 4 Chi-square test for positive rate of 6bacterial species before, during and after COVID-19 pandemic. P0 is before and during COVID-19, P1 is during and after COVID-19, P2 is before and after COVID-19.
