# Correction: Changes in children respiratory infections pre and post COVID-19 pandemic

**DOI:** 10.3389/fcimb.2025.1644555

**Published:** 2025-06-27

**Authors:** Yuanyuan Yue, Dan Wu, Qian Zeng, Yurong Li, Chun Yang, Xin Lv, Ling Wang

**Affiliations:** ^1^ Clinical Laboratory, Children’s Hospital Affiliated to Shandong University, Jinan, China; ^2^ Clinical Laboratory, Jinan Children’s Hospital, Jinan, China

**Keywords:** COVID-19, respiratory infection, non-pharmaceutical interventions, children, qPCR

In the published article, there was an error in **Table 1** as published. The end date was not “January 31, 2014”, it should be corrected to “January 31, 2024”; and in **Table 1**, Under the “Age” column, the category “01” lacks a hyphen. This should be revised to “0-1” for consistency with standard notation. The corrected **Table 1** and its caption appear below.

**Table 1:** The PCR results of 13 pathogens were summarized from January 31, 2018 to January 31, 2024.

year		2018 N=2761 n %		2019 N=22785 n %		2020 N=27337 n %		2021 N=71093 n %		2022 N=113049 n %		2023 N=82332 n %		2024 N=17953 n %		Sum N=337310 n %	
sex	male	1684	60.99	13472	59.13	16224	59.35	41893	58.93	66679	58.98	48093	58.41	10446	58.19	198491	58.85
	female	1077	39.01	9313	40.87	11113	40.65	29200	41.07	46370	41.02	34239	41.59	7507	41.81	138819	41.15
age	0-1	1375	49.80	4511	19.80	5622	20.57	12526	17.62	20786	18.39	10809	13.13	2398	13.36	58027	17.20
	1-3	836	30.28	6577	28.87	8671	31.72	18814	26.46	27703	24.51	18517	22.49	4007	22.32	85125	25.24
	3-6	342	12.39	7304	32.06	8026	29.36	22293	31.36	41574	36.78	29202	35.47	4328	24.11	113069	33.52
	>6	208	7.53	4393	19.28	5018	18.36	17460	24.56	22986	20.33	23804	28.91	7220	40.22	81089	24.04
pathogen	KPN	83	6.01	83	4.53	37	2.76	99	1.24	91	1.49	320	8.84	21	5.48	734	3.24
	PAE	25	1.81	26	1.42	27	2.01	148	1.85	104	1.70	168	4.64	45	11.75	543	2.40
	SA	182	13.19	227	12.38	119	8.87	465	5.83	412	6.73	282	7.79	17	4.44	1704	7.52
	SP	414	30.00	525	28.64	283	21.09	1344	16.84	963	15.72	2205	60.91	290	75.72	6024	26.58
	HIB	354	25.65	546	29.79	256	19.08	1749	21.92	1164	19.00	1008	27.85	138	36.03	5215	23.01
	LP	1	0.07	2	0.11	7	0.52	4	0.05	5	0.08	5	0.14	0	0.00	24	0.11
	MP	62	4.49	1757	16.30	518	5.55	5269	19.93	1375	5.83	258	2.40	125	9.45	9364	11.20
	Flu A	-	-	777	22.14	966	20.69	7	0.13	1727	10.57	2587	18.72	116	3.70	6180	13.13
	Flu B	-	-	143	4.10	674	14.46	1399	25.05	1619	9.91	66	0.48	595	18.96	4496	9.56
	ADV	-	-	303	9.53	170	4.62	450	4.22	1191	7.13	987	7.31	580	18.94	3681	7.25
	RSV	-	-	-	-	298	10.70	868	18.38	1221	7.84	1842	13.41	805	25.65	5034	12.60
	PIV	-	-	-	-	45	5.72	405	4.78	309	5.78	107	2.67	14	0.57	880	4.18
	RhV	-	-	-	-	16	20.25	240	14.65	1474	11.28	1184	13.06	146	11.04	3060	12.16

“n”: Number of positives; “%”: Positive rate.

KPN, Klebsiella pneumoniae; PAE, Pseudomonas aeruginosa; SA, Staphylococcus aureus; SP, Streptococcus pneumoniae; HI, Haemophilus influenzae; LP, Legionella pneumophila; MP, Mycoplasma Pneumoniae; Flu A, Influenza A virus; Flu B, Influenza B virus; ADV, Adenoviridae; RSV, respiratory syncytial virus; PIV, parainfluenza virus; RhV, Rhinovirus; “-”: The test was not performed in our laboratory.“-”

In the published article, there was an error in the **Funding** statement. This research was funded by one Science and Technology Development Program of Jinan Municipal Health Commission, not two. The original text: “The author(s) declare that financial support was received for the research and/or publication of this article. This research was funded by two Science and Technology Development Program of Jinan Municipal Health Commission (No. 2023-1–60 and No. 2023-2-139). All grant numbers and funding information are included in full and accurately.” The correct **Funding** statement appears below.

“The author(s) declare that financial support was received for the research and/or publication of this article. This research was funded by Science and Technology Development Program of Jinan Municipal Health Commission (No. 2023-1-60). All grant numbers and funding information are included in full and accurately.”

In the published article, there was an error. The bacterial name “*Listeria monocytogenes* (abbreviated as LP)” is incorrect. It should be revised to “*Legionella pneumophila* (LP).”

A correction has been made to **Abstract**, *Results:*, From line 81 to line 86. This sentence previously stated:

“A variety of ARIs pathogens, including Influenza A (Flu A), Influenza B (Flu B), Adenovirus (ADV), Rhinovirus (RhV), and Respiratory Syncytial Virus (RSV), as well as co-infecting bacterial such as Klebsiella pneumoniae (KPN), Pseudomonas aeruginosa (PAE), Streptococcus pneumoniae (SP), Haemophilus influenzae (HI), and Listeria monocytogenes (LP), reached a peak positive rate at the age of 3.”

The corrected sentence appears below:

“A variety of ARIs pathogens, including Influenza A (Flu A), Influenza B (Flu B), Adenovirus (ADV), Rhinovirus (RhV), and Respiratory Syncytial Virus (RSV), as well as co-infecting bacterial such as Klebsiella pneumoniae (KPN), Pseudomonas aeruginosa (PAE), Streptococcus pneumoniae (SP), Haemophilus influenzae (HI), and Legionella pneumophila (LP), reached a peak positive rate at the age of 3.”

The authors apologize for this error and state that this does not change the scientific conclusions of the article in any way. The original article has been updated.

