# Supplementary material for: Changes in children respiratory infections pre and post COVID-19 pandemic
Source: Front Cell Infect Microbiol. 2025 Apr 7;15:1549497. doi: 10.3389/fcimb.2025.1549497 (PMC12009837; doi:10.3389/fcimb.2025.1549497)
Supplement: Supplementary Table 3 — Chi-square test for positive rate of 7 infectious pathogens before and after COVID-19 pandemic. [file Table3.docx]

| pathogen | during COVID-19（2020-2022）  n % | | post-COVID-19（2023-2024.1）  n % | | P |
| --- | --- | --- | --- | --- | --- |
| MP | 7162 | 12.07% | 383 | 3.17% | <0.001 |
| Flu A | 2700 | 10.15% | 2703 | 15.94% | <0.001 |
| Flu B | 3692 | 13.89% | 661 | 3.90% | <0.001 |
| ADV | 1811 | 5.84% | 1567 | 9.46% | <0.001 |
| RSV | 2387 | 10.34% | 2647 | 15.68% | <0.001 |
| PIV | 759 | 5.19% | 121 | 1.88% | <0.001 |
| RhV | 1730 | 11.70% | 1330 | 12.80% | 0.009 |

1. **Supplementary data table 3：Chi-square test for positive rate of 7 infectious pathogens before and after COVID-19 pandemic.**

| pathogen | pre-COVID-19  （2018-2019）  n % | | during COVID-19  (2020-2022)  n % | | P0 | post-COVID-19  (2023-2024)  n % | | P1 | P2 |
| --- | --- | --- | --- | --- | --- | --- | --- | --- | --- |
| KPN | 166 | 5.17% | 227 | 1.47% | <0.001 | 341 | 8.52% | <0.001 | <0.001 |
| PAE | 51 | 1.59% | 279 | 1.81% | 0.392 | 213 | 5.32% | <0.001 | <0.001 |
| SA | 409 | 12.73% | 996 | 6.45% | <0.001 | 299 | 7.47% | 0.021 | <0.001 |
| SP | 939 | 29.23% | 2590 | 16.77% | 0.000 | 2495 | 62.33% | 0.000 | <0.001 |
| HI | 900 | 28.01% | 3169 | 20.52% | <0.001 | 1146 | 28.63% | <0.001 | 0.563 |
| LP | 3 | 0.09% | 16 | 0.10% | 0.869 | 5 | 0.12% | 0.714 | 0.689 |

1. **Supplementary data table 4：Chi-square test for positive rate of 6bacterial species before, during and after COVID-19 pandemic. P0 is before and during COVID-19，P1 is during and after COVID-19，P2 is before and after COVID-19.**
